# The Effect of Valeric on Anxiety Severity in Women Undergoing Hysterosalpingography

**DOI:** 10.5539/gjhs.v7n3p358

**Published:** 2015-04-02

**Authors:** Maryam Gharib, Leila Neisani Samani, Zahra Elahi Panah, Mohsen Naseri, Naser Bahrani, Kiandokht Kiani

**Affiliations:** 1 Tehran University of Medical Sciences, Tehran, Iran; 2 Iran University of Medical Sciences, Tehran, Iran; 3 Radiology Group, Tehran University of Medical Sciences, Tehran, Iran; 4 Clinical Trial Research Center for Traditional Medicine, Shahed University, Tehran, Iran; 5 Shahid Sattari University of Aeronautical Engineering, Iran; 6 Royan Institute, Iran

**Keywords:** valeric, hysterosalpingography, anxiety

## Abstract

**Aim and Scope:**

Hysterosalpingography (HSG) is the radiographic evaluation of the uterine cavity and fallopian tubes, which is generally assumed as a stressful and painful procedure. This study aims to determine effect of oral Valeric capsules on anxiety severity in women under Hysterosalpingography.

**Method and Examination:**

this study, as a double-blind clinical trial, was conducted on 64 infertile women undergoing hysterosalpingography, who referred to radiology ward at Comprehensive Women’s hospital. To measure anxiety, visual analog anxiety scale was used 90 minutes before starting procedure, individuals in intervention group (n=32) received a single dose (1,500 mg) of 3 Valeric capsules, together with routine prophylaxy, where routine prophylaxis contains Mefenamic acid 250 mg capsules in 30 minutes before procedure, and the same capsules were prescribed to placebo group (n=32) with the same instruction. Anxiety severity before and once 90 minutes after intervention in both groups were measured and compared.

**Results:**

There was no difference on anxiety severity before intervention in both groups (p=0.26), and the groups were homogeneous; after intervention, a significant difference on anxiety severity was reported in both groups (p<0/0001), and anxiety score in intervention group compared to placebo reduced statistically.

**Conclusion:**

Present study indicated that Valeric was effective on reducing anxiety in women undergoing hysterosalpingography.

## 1. Introduction

In industrial countries, about 6.6 to 26.4percent of couples at reproductive age are infertile, and the potential reasons for infertility in women go beyond, including disorder at any fallopian tubes, uterus, cervix and ovaries, but more specifically a disorder in fallopian tubes is the reason for infertility for about 30 to 40 percent. Hysterosalpingography is a part of common diagnostic examinations for infertility, however development of other diagnostic instruments such as MRI radiography has been largely seen, hysterosalpingography has been considered as a standard instrument for evaluation of openness of fallopian tubes (La Fianza et al., 2014). Infertile couples during management and treatment of infertility, recurrent abortion, sometimes adverse consequences of pregnancy oblige to undergo invasive diagnostic procedures (16). Invasive imaging techniques relating to the reproductive system are mainly followed by various degrees of anxiety and concern, pain and physical damages in women, mentioned that hysterosalpingography is followed by states of severe anxiety before and during procedure (DerMarderosian & Beutler, 2002). In recent decades, stress management during uncomfortable medical procedures has been drawn into attention in order to making it tolerable for the patients According to various studies, level of anxiety in women undergone hysterosalpingography increases, and it can influence patient’s assumption from pain and medical procedure as well as physician’s performance during procedure. Anxiety can influence results and complications from medical invasive techniques, where these complications include bleeding, pelvic infection, uterine perforation, cervical laceration and uterine cramps and laceration of the cervix (16). In a study, anxiety in women undergoing three different imaging types including Hysterosalpingography, abdominal ultrasonography, and mammography was examined, and the results indicated that women undergoing Hysterosalpingography obviously suffered from high level of anxiety rather than abdominal ultrasonography and mammography. This indicates that a level of anxiety before and during Hysterosalpingography is considered in women undergoing Hysterosalpingography (DerMarderosian & Beutler, 2002). A variety of studies in the context of reduction of anxiety in such procedures using non-drug therapies and techniques have been conducted. Agon et al. (2005) examined effect of music on anxiety in patients undergoing Hysterosalpingography, and the results indicated that physical indicators of anxiety in test group had reduced rather than control group (Burke Jonathan, 2007). In a study by Alferd et al. (2014) entitled “a study on effect of education and single counseling on reduction of anxiety in women undergoing hysterosalpingography”, the results indicated effectiveness of education and single counseling as an intervention in reducing anxiety signs among women undergoing hysterosalpingography (La Fianza et al., 2014). Complementary medicine is one of the approaches for anxiety treatment, where science and experience have proven that medicinal herbs have therapeutic properties. Currently, some developed countries have provided the drugs derived from herbs which they often use them, so far as over 80% of people around the world use 200 species of common medicinal herbs throughout the world for treatment of various diseases, based on World Health Statistics. In this regard, according to the statistics in Iran, in recent years traditional medicine and use of medicinal herbs have been welcomed more. Among medicinal herbs used in various contexts, cinnamon, chamomile, valerian and green tea are those medicinal herbs that can be used for anxiety disorders (Mirabe, Dolatian, Mojab, & Namdari, 2012). Valerian, in pharmacology and herbal medicine, is widely used as an anti-anxiety or a treatment for insomnia, so that some of botanists know sedative and/or anxiolytic action of valerian extracts as Valerian drug target (Yang et al., 2012). Valeric, existing acid in Valerian root, is effective in reducing anxiety and increasing quality of sleep by affecting level of serotonin and noradrenaline; further, Valepotriates causes increasing sleep and relief of pain, and the products from analysis of it such as Baldrinal and Homobaldrinal cause reducing anxiety signs. The highest effect of valerian include increasing GABA levels, where treatment of anxiety can be acquired by reducing vagus nerve stimulation through increasing GABA level (Fatma, Aysegul, & Yasemin, 2012). Valerain consisting of Valepotriates and Valeric acid has been connected to Valium-like receptors in the brain, and it can be used as tablet, antispasmodic, anxiety and sleep disorders (Agwn & Okoye, 2005). According to clinical studies through extract of this herb, not complication, unwanted or side effects from have been reported. This herb has been recognized in group A in Australia, and it has been allowed to be used in food products by FDA in USA (Mehdi, 2012; Robert, Chaplin Jr., David, Donna, Lawrence, & Normalynn, 2007; Samsam, 2007). It seems that due to such biochemical properties, Valerian enable to be effective in inhibiting anxiety in individuals undergoing Hysterosalpingography. According to our examinations, a study in this context has not been conducted abroad or inside country, but drug information websites, books on medicinal plants and some articles have known use of this herb effective in treatment of spasms, headaches, nervous symptoms, anxiety and stress. Hence, according to abundant diagnostic-therapeutical application of this procedure in infertility examinations, regarding therapeutical effect of Vlerian in the context of Anxiolytic properties of this herb proven in various studies, the researchers decided to conduct a study aiming at determining effect of valeric tablet on anxiety severity in women undergoing hysterosalpingography.

## 2. Materials and Methods

This study confirmed by ethics committee of the Medical University of Tehran has been conducted. This study as a double-blind clinical trial was conducted on 64 infertile women undergoing Hysterosalpingography, who referred to radiology ward at Comprehensive Women’s Hospital & Health Centre. To measure anxiety, visual analog anxiety scale was used. 90 minutes before starting procedure, individuals in intervention group (32 individuals) received 3 Valeric capsules 500 mg in a single dose (1,500 mg) together with routine prophylaxis, where routine prophylaxis contains Mefenamic acid 250 mg capsules in 30 minutes before procedure, and the same capsules were prescribed to placebo group (32 individuals) with the same instruction. Anxiety severity before intervention and once 90 minutes after intervention in both groups were measured and compared. Inclusion criteria include lack or suspicious pregnancy, no history of mental and physical illness, lack of experience for Hysterosalpingography and effective drugs for pain and anxiety, litterate, no history of drug senility and allergy, no contraindications of Hysterosalpangiography including pelvic inflammatory disease (PID) or cervicitis and no history of uterine surgery. The sample size regarding the same studies was estimated at 95% confidence level and standard error (5%) and affect size of (0.8). Firstly the aim of research and how the research will be conducted, were defined for patients, and consent form was given to them. In this study, data collection instruments include demographic questionnaire and visual analog scale. Visual analog scale was used to measure anxiety. However, questionnaire or interviews are the common methods to measure anxiety; visual analog scale has been used in some studies as well as this study to measure anxiety, where it has been used due to study conditions to measure severity of anxiety in this study. Demographic questionnaire includes the information such as age, ethnicity, education level of the couples, residency, and employment status of couples, socioeconomic status, and duration of infertility. in addition to validity and reliability, the ease to use visual analog scale is the most important feature of this instrument, where Visual analog scales (VAS) ranging from 0 cm (no anxiety) to 10 cm (worst imaginable anxiety) are used widely for anxiety measurement; acquiring score 1-3, 4-7 and 8-10 represents mild anxiety, moderate anxiety and severe anxiety, respectively. Visual analog scale is a standard instrument confirmed by researchers and scholars for several time, further content and face validity of this instrument using views of 10 faculty members in Nursing and Midwifery Faculty of Medical Science University and Medical Health Center of Tehran, were confirmed. To ensure qualified participants attending in the study, firstly a personal attributes questionnaire was filled and then a medical history for person’s health was also filled. In next stage, a simple random sampling method has been considered, such that drugs in form of cards C and D were provided for research unit. The first person voluntary selected one of codes, where code C was selected, and then the rest of units under research were considered in two groups C and D one by one. Blindness was in this way that the capsules in two groups were in yellow-brown colors provided in 3 capsules in similar packs with codes C and D, where the researcher and samples was not informed of type of drug and just codes C and D were clear. Drugs were provided by clinical trial research center for traditional medicine-Shahed University in form of drug and placebo in form of uniform capsules 500 mg in a single dose (1500 mg) with codes C and D. intervention group received 1500 mg valeric tablet 90 minutes before intervention together with a glass of warm water, in addition to 30 minutes before intervention with routine prophylaxis containing 250 mg Mefenamic acid. Control group also received 1500 mg placebo containing rice flour, using the same instruction for intervention group. How anxiety evaluated was in this way that firstly anxiety level was asked via visual analog scales before consuming the drug, and then it was measured by the researcher 90 minutes after intervention and before Hysterosalpingography, and recorded. In this study, to analyze data, SPSS Software version 22 was used. Descriptive statistical methods including frequency distribution table, mean and standard deviation were used to define personal traits of individuals under study.

## 3. Results

According to Table 1, traits of age and women’s infertility period in terms of mean and standard deviation have been represented, where there was no significant difference on age and women’s infertility in two groups, and both groups were homogenous. Age of a majority of women was in age group 30-40 years old (50% placebo, 46.87% of the intervention group), and their education status was diploma (4.4% placebo group, 56.3% the intervention group), and their employment status was housewifery (81.3% placebo group, 71.9% the intervention group). Average economic status was seen more in majority of women (68.8% in the placebo group, 90.6% intervention group), and their residency was in city (90.6% in the placebo group, 96.6% in the intervention group). Determination and comparison of anxiety score before intervention (90 minutes to hysterosalpingography) between intervention and control groups have been represented in Table 2. Mean and standard deviation of anxiety 90 minutes before intervention in intervention and placebo groups have been in turn equal to 5.5±2.66 and 4.9±2.83. Results of Mann-Whitney test indicated that there was no significant difference on mean of anxiety scores in 90 minutes before intervention in two groups (p=0.26). Further, results of Chi-square test indicated that majority of women in placebo group before intervention enjoyed average anxiety severity (43.8) and majority of women in intervention group before intervention enjoyed severe anxiety severity (40.6). Determination and comparison of anxiety score before intervention and hysterosalpingography between intervention and control groups have been represented in Table 2. Mean and standard deviation of anxiety 90 minutes after intervention in intervention and placebo groups have been in turn equal to 3.5±2.28 and 6.03±2.50. Results of t-test indicated that there is a significant difference on mean of anxiety scores in two groups after intervention, and mean of scores in intervention group compared to placebo group indicated a significant decrease.

**Table 1:** Traits of age and infertility duration in terms of mean and standard deviation in two intervention and placebo groups

Group	Intervention Group		Placebo Group		Result of t-test
	Standard deviation	Mean	Standard deviation	Mean	
age	**7/50**	**30/6**	**6/42**	**32/4**	P-value=0/64
infertility duration	**2/21**	**2/9**	**2/59**	**2/9**	P-value=0/86

**Table 2:** comparison of mean of anxiety scores before intervention and mean of anxiety scores after intervention in two intervention and placebo groups

Group	intervention group		placebo group		Result of test
	Standard deviation	Mean	Standard deviation	Mean	
Anxiety in 90 minutes before intervention	2/26	**5/5**	2/83	**4/9**	Mann-Whitney test p-value=0/26
Anxiety in 90 minutes after intervention	2/28	**3/5**	2/50	**6/03**	Mann-Whitney test pvalue<0/0001

## 4. Discussion

This medical trail aimed to determine effect of Valeric capsules on anxiety severity in women undergoing Hysterosalpingography. A significant difference on anxiety scores between intervention and placebo groups was seen. Results of statistical test indicated that there was no difference on anxiety scores mean in 90 minutes before intervention in both groups, while anxiety severity before intervention was reported ranging from average to severe in them. Findings of this study has been relevant with the studies conducted in past; according to the study by Alferd et al. (2014) entitled “Effectiveness of a Single Education and Counseling Intervention in Reducing Anxiety in Women Undergoing Hysterosalpingography”, there was no difference on clinical signs before intervention in both groups, and the groups were homogenous. Further, there was no difference on awareness level from Hysterosalpingography between groups (La Fianza et al., 2014). In a study by Haji-Hassan et al. (2012) entitled “Assessment Relaxation Effect on Anxiety in Infertile Women undergoing Assisted reproductive techniques during induction ovulation”, anxiety level in research units has been in average level from the starting study, and the findings of this study have been relevant with other studies (Tori Hudson, 2003).

In a study that Yang et al. (2012) have examined effect of education and counseling intervention in the individuals under invasive procedure of Carotid endarterectomy, higher level of anxiety was reported in the groups under study before counseling intervention (Valle-Mojica, Ayala-Marín, Ortiz-Sanchez, Torres-Hernández, Abdalla-Mukhaimer, & Ortiz, 2011).

Fatma Gül AKSOY et al. (1999) in a study examined situational anxiety in women under three types of radiological procedures including Hysterosalpingography, abdominal ultrasonography, and mammography that each in turn had various degrees of mild, moderate and high levels of physical and psychological discomfort; the results indicated that the women under Hysterosalpingography obviously have higher level of situational anxiety compared to two other procedures. This implies that a level of anxiety before and after procedure is considered in women under Hysterosalpingography (Der Marderosian & Beutler, 2002). To compare effect of intervention on anxiety severity, it requires the same level of anxiety in both groups. In this study, there was a significant difference on mean of anxiety scores in 90 minutes after intervention in both groups, a significant decrease was seen in mean of scores in test group rather than control group (p<0.0001). Various studies in the context of decrease of anxiety in diagnostic-therapeutic procedure of Hysterosalpingography using non-drug procedures have been conducted.

Agwu et al. (2005) examined the effect of music on the anxiety levels of women undergoing hysterosalpingography; the results of this study indicated that physical indicators of anxiety in test group rather than control group had reduced (Burke Jonathan, 2007). Furthermore, according to the study by Alferd et al.(2014), the results from study indicated effectiveness of education and counseling intervention as an intervention in reducing anxiety signs among women undergoing Hysterosalpingography (La Fianza et al., 2014). According to the study by Yang et al. (2012), anxiety level was shown with a significant decrease in intervention group rather than control group (Valle-Mojica et al., 2011). The present study reported the effective role of complementary and alternative medicine in reducing anxiety level in invasive procedure of Hysterosalpingography.

Robert et al. (2007) announced that valerian can be widely used as for anti- anxiety or insomnia treatment, so that some of botanists know the feature of valerian for anti- anxiety or insomnia treatment as valerian drug target (Yang et al., 2012).

In the study by Lisa et al. (2011), Valerenic acid existing in valerian by affecting levels of serotonin and noradrenaline can be effective in reducing anxiety and increasing quality of sleep (Fatma et al., 2012). Valepotriates also cause increasing sleep and relief of pain, and the products from analysis of Valepotriates such as such as Baldrinal and Homobaldrinal cause reducing anxiety signs. The highest effect of valerian include increasing amount of Gamma Amino Butiric Asid through which treatment of anxiety by reducing vagus nerve stimulation will be obtained through increasing amount of gaba (Fatma et al., 2012).

Valerian with the scientific name of Valeriana officinalis belongs to Valerianaceae species, which has been used as a herbal drug since century 11 for pain relief, and it has been used since century 16 as a sedative herb in France, Germany and Switzerland (Mirabe, Dolatian, Mojab, & Alavi Majd, 2009). Valerain consisting of valepotriates and Valeric acid has been connected to Valium-like receptors in the brain, and it can be used as tablet, antispasmodic, anxiety and sleep disorders (Agwn & Okoye, 2005). According to clinical studies through extract of this herb, unwanted effects or side effects from this herb have been reported. This herb has been recognized in group A in Australia, and it has been allowed to be used in food products by FDA in USA (Mehdi, 2012; Robert et al., 2007; Samsam, 2007). Further, in this study, effect of valeric tablet on anxiety severity in women undergoing hysterosalpingography has been largely seen, reducing anxiety in intervention group rather than another group. Then, valeric tablet as a complementary and alternative medicine can be effective in controlling anxiety of individuals undergoing hysterosalpingography.

## 5. Conclusion

The present study indicated that Valeric causes reducing anxiety in women undergoing HSG considerably. Therefore, it is suggested to prescribe Valeric capsules in HSG and examine in other invasive imagimg procedures on internal female reproductive system.
